# Nitrogen Application Rate Affects the Accumulation of Carbohydrates in Functional Leaves and Grains to Improve Grain Filling and Reduce the Occurrence of Chalkiness

**DOI:** 10.3389/fpls.2022.921130

**Published:** 2022-06-24

**Authors:** Changchun Guo, Xiaojuan Yuan, Fengjun Yan, Kaihong Xiang, Yunxia Wu, Qiao Zhang, Zhonglin Wang, Limei He, Ping Fan, Zhiyuan Yang, Zongkui Chen, Yongjian Sun, Jun Ma

**Affiliations:** ^1^Rice Research Institute, Sichuan Agricultural University, Chengdu, China; ^2^Crop Ecophysiology and Cultivation Key Laboratory of Sichuan Province, Chengdu, China

**Keywords:** nitrogen, carbohydrate, dynamic accumulation, grain-filling, chalkiness

## Abstract

Chalkiness, which is highly affected by nitrogen (N) management during grain filling, is critical in determining rice appearance quality and consumer acceptability. We investigated the effects of N application rates 75 (N_1_), 150 (N_2_), and 225 (N_3_) kg ha^−1^ on the source-sink carbohydrate accumulation and grain filling characteristics of two *indica* hybrid rice cultivars with different chalkiness levels in 2019 and 2020. We further explored the relationship between grain filling and formation of chalkiness in superior and inferior grains. In this study, carbohydrates in the functional leaves and grains of the two varieties, and grain filling parameters, could explain 66.2%, 68.0%, 88.7%, and 91.6% of the total variation of total chalky grain rate and whole chalkiness degree, respectively. They were primarily concentrated in the inferior grains. As the N fertilizer application rate increased, the chalky grain rate and chalkiness degree of both the superior and inferior grains decreased significantly. This interfered with the increase in total chalky grain rate and chalkiness. Moreover, the carbohydrate content in the functional leaves increased significantly in N_2_ and N_3_ compared with that in N_1_. The transfer of soluble sugar from the leaves to the grains decreased the soluble sugar and increased total starch contents, accelerated the development of grain length and width, increased grain water content, and effectively alleviated the contradiction between source and sink. These changes promoted the carbohydrate partition in superior and inferior grains, improved their average filling rate in the middle and later stages, optimized the uniformity of inferior grain fillings, and finally led to the overall reduction in rice chalkiness.

## Introduction

As one of the major food staples, rice provides the essential carbohydrate demand for nearly 65% of the population globally (Cantrell and Reeves, 2002; Rao et al., 2014). However, frequent regional or seasonal climate change, rapid population increase, and improvement in living standards have posed significant challenges to the production of high-quality rice (Zhou et al., 2015; Deng et al., 2018; Chen et al., 2021). Therefore, improving rice quality is urgently required.

Rice quality parameters mainly include processing, appearance, cooking, eating, and nutritional quality (Li et al., 2016; Zhu et al., 2017). Among them, appearance is widely considered as one of the critical factors affecting the acceptance of rice by consumers. Chalkiness is the primary index to evaluate the appearance quality of rice. It is not easy to overcome this challenge in rice production (Peng et al., 2014; Yu et al., 2017). Deficient amyloplast and protein development in the endosperm and increased interspaces between the loosely packed starch granules lead to an increase in chalkiness and an overall decline in rice quality (Lisle et al., 2000; Singh et al., 2003; Ishimaru et al., 2009). Its location on or within the endosperm can be categorized into white-belly, white-core, white-back, etc. (Tashiro and Wardlaw, 1991; Ishimaru et al., 2009). At present, evidence suggests that cultivation factors, such as nitrogen (N) application and high temperature, have a significant effect on chalkiness formation (Zhou et al., 2015; Shi et al., 2017; Wada et al., 2019; Chang et al., 2021; Yang et al., 2021; Sun et al., 2022).

N fertilizer is an essential agronomic measure that affects rice growth, yield, and quality (Gu et al., 2015, 2017). An appropriate amount of N fertilizer produces a series of changes in the external morphology and internal physiological characteristics of rice. They include enhancing root activity, leaf color, net photosynthetic rate, nutrient absorption and transport, and the accumulation and redistribution of dry matter weight, which finally results in a marked increase in grain yield (Sun et al., 2012; Zhao and Fitzgerald, 2013; Wei et al., 2017). However, the key role of N fertilizer in rice production is reflected not only in yield but also in rice quality. Rice quality is determined by the starch and protein storage ratio produced by carbon (C) and N metabolism during grain filling. A previous study reported that N application significantly increased the protein content of rice but decreased amylose content (Zhou et al., 2020). The changes in these substances are related to the supply of external cultivation conditions. Previous studies have shown that N application improves the sucrose produced by leaf photosynthesis or starch degradation. These are transported to the storage organ (grain) through the phloem, affecting the speed of grain filling, leading to the increase or decrease in rice chalkiness (Wei et al., 2018; Huang et al., 2019). To date, several studies have investigated the mechanism of rice chalkiness formation from the perspectives of endosperm cell development and nutrient transport, relationship between source and sink supply, and molecular genetic mechanism, and they preliminarily proved that the processes of endosperm cell development, leaf photosynthetic product and grain nutrient input have a significant impact on the development of rice chalkiness (Raju and Srinivas, 1991; Zheng et al., 2012; Xi et al., 2014). In addition, research on the mechanism of chalkiness formation mostly focuses on the activities of key enzymes of carbon metabolism and the accumulation of starch, while studies on grain N metabolism and its product, protein, are relatively lacking. Rice chalkiness is a result of combined action of grain carbon and N metabolism. N metabolism provides the key enzymes for carbon metabolism, while carbon metabolites provide the carbon skeleton and energy necessary for N metabolism. This provides direction to reveal the response of rice chalkiness to N fertilizer. However, in Southwest China, the physiological mechanism underlying decrease in rice chalkiness due to the N fertilizer rate is not fully elucidated.

Therefore, our field experiments were performed using three N fertilizer gradients and two hybrid *indica* rice varieties with different chalkiness characteristics. The objectives of this study were to: (a) investigate the effects of N application rate on the accumulation of carbohydrates in the functional leaves and caryopsis, grain filling and chalkiness on different chalky rice varieties and (b) assess the relationship between grain filling parameters and the formation of superior and inferior grains chalkiness. We aimed to explore the mechanism of chalkiness formation under different N application rates and provide practical strategies for high-quality rice production.

## Materials and Methods

### Experimental Sites and Plant Materials

**Figure 1:**
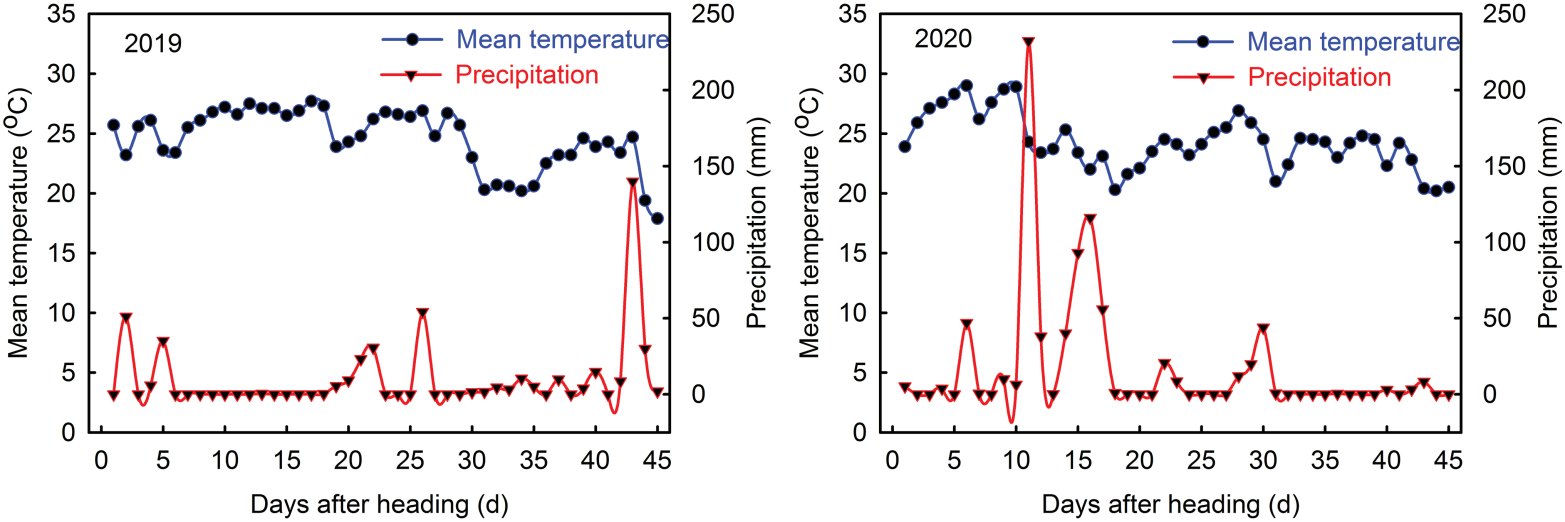
Mean temperature and precipitation during rice grain-filling from Wenjiang experimental field in 2019 and 2020.

The field experiment was carried out at Sichuan Agricultural University (103°47′ E, 30°43′ N, and 536.0 m altitude), Sichuan Province, China, from April to October 2019 and 2020. The study site is located in the Chengdu Plain and has a subtropical humid climate, with average annual rainfall of 798.3–1541.0 mm, average annual temperature of 15.7°C–17.7°C, and average annual sunshine of 685.5–1002.9 h. The soil texture is sandy loam, with a robust water-holding capacity and is suitable for rice cultivation. Meteorological data during the basal nutrient content and grouting of farming layer soil are shown in Table 1 and Figure 1. The study material included *indica* three-line hybrid rice cultivars, Chuannongyou 508 (C_1_) with high chalkiness and Shuangyou 573 (C_2_) with low chalkiness. These cultivars were bred by the Rice Research Institute of Sichuan Agricultural University. To ensure that all varieties received the same temperature and light conditions after heading, during the 2 years, the sowing dates of the two varieties were the same, C_1_ was manually seeded on April 19 and C_2_ on April 26.

**Table 1:** Physicochemical characteristics of soil conditions in rice plow layer (0–20 cm) from Wenjiang experimental field in 2019 and 2020.

Year	Total N (g kg^−1^)	Organic matter (g kg^−1^)	Available N (mg kg^−1^)	Available P (mg kg^−1^)	Available K (mg kg^−1^)
2019	1.53	21.30	101.31	28.49	86.32
2020	1.32	19.76	92.75	28.15	83.33

### Experimental Design and Field Management

The experiment adopted a two-factor split-plot design, with N fertilizer as the main plot and *indica* hybrid rice cultivars as the split-plot. There were six treatments, repeated thrice, totaling 18 plots. The plot area was 15 m^2^ (5 m × 3 m), sowing with row and plant spacing of 25 cm and 20 cm, with 300 holes (12 rows and 25 holes per row) in each plot. Four to six seeds were sown in each hole. After emergence, two seedlings were fixed per hole.

The application gradients of N fertilizer (urea) were 75 (N_1_), 150 (N_2_), and 225 (N_3_) kg ha^−1^, respectively. N was applied three times in the whole growth period, 40% on basal (7 days after sowing), 30% on tillering (25 days after sowing), and 30% on panicle initiation stage (70 days after sowing). Phosphate (P_2_O_5_ 75 ka ha^−1^) was applied only as a basal application. Potassium (K_2_O 150 kg ha^−1^) was applied in the same amount at the basal and panicle initiation stages. A 30 cm ridge was built between the plots and covered with plastic film to prevent mutual leakage of water and fertilizer between plots. The plots were regularly weeded and treated with insecticides.

### Determination Items and Methods

#### Field Panicle Marker and Sample Preparation

Four hundred single panicles with the same heading size and flowering time were selected for each plot and marked with red tags at the heading stage. Fifteen single panicles with functional leaves (top three leaves) were collected from each plot at 6, 12, 18, 24, 30, 36, and 42 days after anthesis (DAA). The grains from the first, second, and third grain positions from the bottom to top in the primary and secondary branches of the panicles were picked. The functional leaves and collected grains were placed in a ventilated oven (XMTD-8222, Shanghai Hongjing Test Equipment Factory, China) at 105°C for 30 min and then dried at 80°C to a constant weight. After grain shelling (JLGJ 4.5, Taizhou Cereal and Oil Instrument Co., Ltd., Zhejiang, China), the leaves and grains (caryopsis) were crushed and sieved separately as reserve.

#### Determination of Carbohydrates in Functional Leaves and Grains

The contents of soluble sugar, sucrose, and starch in leaves and grains were determined using the method described by DuBois et al. (1956) and Zhang (1977) with slight modifications. The sugar test liquid extraction was done as follows: 0.1 g sample was extracted with 5.0 ml of 80% ethanol at 80°C for 30 min. After repeated extraction and centrifugation (6,000 ×*g* for 5 min) three times, the supernatant was combined. Then, 50 mg activated carbon was added to the supernatant and decolorized at 80°C for 30 min. The decolorization solution (testing solution) was collected, and the volume was adjusted to 25 ml with 80% ethanol to analyze soluble sugar, sucrose, and starch content. A detailed method of measuring fructose and glucose contents is provided in Supplementary Figures 1 and 2.

The soluble sugar content was analyzed by taking 1.0 ml of the testing solution and adding to 2.0 ml of distilled water and 5 ml of chromogenic agent (1 g of anthrone dissolved in 1000 ml of dilute sulfuric acid) and keeping it in a water bath at 100°C for 10 min. After cooling, the absorbance was measured at 620 nm.

The sucrose content was analyzed by taking 0.5 ml of testing solution and mixing it with 100 μl 2 M NaOH. The solution was kept in a boiling water bath for 10 min to remove the reducing sugar. After that, 3.5 ml of 30% HCl and 1 ml of 0.1% resorcinol were added to the sample and kept in a water bath at 80°C for 10 min. The absorbance was then measured at 480 nm.

The starch content was determined by using the precipitate after extraction of the sugar, mixing it with 2.0 ml water, and gelatinizing it at 100°C for 15 min. After cooling, 2 ml of 9.2 M perchloric acid and 2 ml distilled water were added to the sample. Extracted for 15 min, and centrifuged at 6,000 *g* for 5 min. The supernatant was collected. Exactly 2 ml of 4.6 M perchloric acid and 4 ml distilled water were added to the residual precipitation, and the previous extraction and centrifugation steps were repeated. The supernatant was combined, and the volume was adjusted to 25 ml with distilled water. Precisely 0.5 ml of anthrone reagent and 5 ml of concentrated sulfuric acid were added to 1 ml of extracting solution and kept at 90°C for 5 min. The absorbance was measured at 620 nm.

#### Analysis of Rice Grain Shape, Water Content, and Grain-Filling Parameter Prediction

In each plot, from the flowering to maturity stage of rice, 12 rice panicles with relatively consistent flowering time and size were marked with red tags and were cut with scissors every 4 days. Superior and inferior grains were obtained during each sampling. The unfertilized empty grains in each sample were removed. After manually removing the rice husk of the remaining grains, the fresh weight was obtained (*M*_1_). The length and width of the superior and inferior grains were measured with a vernier caliper and dried to constant weight, and weighed (*M*_2_). Superior and inferior grains were categorized according to the following methods: on the three primary branches at the top of the panicle, except the second grain, the other grains were superior grains; and on the secondary branches of the three primary branches at the bottom of the panicle, except for the grain at the top of the secondary branch, the other grains were inferior grains. The calculation formula of grain water content and its corresponding figure are shown in Supplementary Figure 3.

Richards’ equation was used to fit the grain filling process with reference to the methods of Sun et al. (2018) and Yang et al. (2003):


(1) $$W=A/{\left(1+Be^{-kt}\right)}^{1/N}$$

where *W* is the weight of a kernel (mg); *A* is the final weight of a kernel; *t* is the time days after anthesis (d); and *B*, *k*, and *N* are equation parameters. *R*^2^ is the fitting degree of the equation. The equation of grain filling rate *G* (mg·kernel^−1^·d^−1^) was obtained by taking the derivative of equation (1):


(2) $$G=AkBe^{-kt}/N{\left(1+Be^{-kt}\right)}^{\left(N+1\right)/N}$$

According to the above equation parameters, we performed the following calculations:

- R_0_ (initial growth potential of grain) =k/N
- T_max_ (days required for grains to reach the maximum filling rate) $=\left(\ln B-\ln N\right)/k$
- W_max_ (weight of a kernel at maximum filling rate) $=A{\left(N+1\right)}^{-1/N}$
- GR_mean_ (average grain filling rate during the whole filling stages) $=Ak/2\left(N+2\right)$
- The whole grain filling period was divided into early (0–*T*_1_), middle (*T*_1_–*T*_2_), and late (*T*_2_–*T*_99_) three stages, respectively.

$$T_{1}=-\ln\left[\left(N^{2}+3N+N\cdot \sqrt{N^{2}+6N+5}\right)/2B\right]/k\text{,}$$

$$T_{2}=-\ln\left[\left(N^{2}+3N+N\cdot \sqrt{N^{2}+6N+5}\right)/2B\right]/k\text{,}$$

$$T_{99}\left(\mathrm{effective filling time}\right)=-\ln\left\{\left[{\left(100/99\right)}^{N}-1\right]/B\right\}/k$$

f. *W*_1_, *W*_2_, and *W*_99_ correspond to the weight of brown rice at *T*_1_, *T*_2_, and *T*_99_ respectively, so the mean filling rate in the early (*MGR_e_*), middle (*MGR_m_*) and late (*MGR_l_*) stage of grain filling was calculated as follows:

$$MGR_{e}=W_{1}/T_{1}\text{,}$$

$$MGR_{m}=\left(W_{2}-W_{1}\right)/\left(T_{2}-T_{1}\right)\text{,}$$

$$MGR_{l}=\left(W_{99}-W_{2}\right)/\left(T_{99}-T_{2}\right)\text{.}$$

g. The contribution rate (RGC) of the accumulation of grain filling materials at each stage to the final grain weight (A) is calculated *via* the following equations:

$$RGC_{e}\left(\%\right)=W_{1}/A\times 100\%\text{,}$$

$$RGC_{m}\left(\%\right)=\left(W_{2}-W_{1}\right)/A\times 100\%\text{,}$$

$$RGC_{l}\left(\%\right)=\left(W_{99}-W2\right)/A\times 100\%\text{.}$$

### Determination of Chalky Grain Rate and Chalkiness Degree

One hundred and fifty labeled panicles were sampled at harvest from each plot, manually divided into superior and inferior grains, screened, and the impurity was removed. Superior and inferior grains were stored under-ventilated conditions for 2 months for further use. After that, they were shelled (JLGJ 4.5, Taizhou Cereal and Oil Instrument Co., Ltd., Zhejiang, China) and milled (JNM-III, China Grain Reserve Chengdu Research Institute Co., Ltd., Chengdu, China). The method described by Sun et al. (2022) and Yoshioka et al. (2007) was used to measure the chalky grain rate and chalkiness of superior and inferior grains with some modifications. Exactly 1,000 whole milled rice were counted from each sample and placed on the scanner to create a digital image. The process was repeated three times. The chalky grain rate and chalkiness degree were obtained through the image analysis software (JMWT-12, Dongfujiuheng Instrument Technology Co., Ltd., Beijing, China). The total chalky grain rate is equal to the sum of the chalky grain rate of superior and inferior grains, and the total chalkiness degree is equal to the sum of the chalkiness degree of superior and inferior grains.

### Statistical Analysis

ANOVA was performed with Statistix 17.0 (SPSS Inc., Chicago, IL), using the least significant difference test at a probability level of 0.05 (*n* = 3). The figures were plotted using SigmaPlot 12.0 (Systat Software Inc., San Jose, CA). Principal component analysis was completed with origin 2021 (OriginLab Corp., Northampton, MA).

## Results

### Chalky Grain Rate and Chalkiness Degree

**Figure 2:**
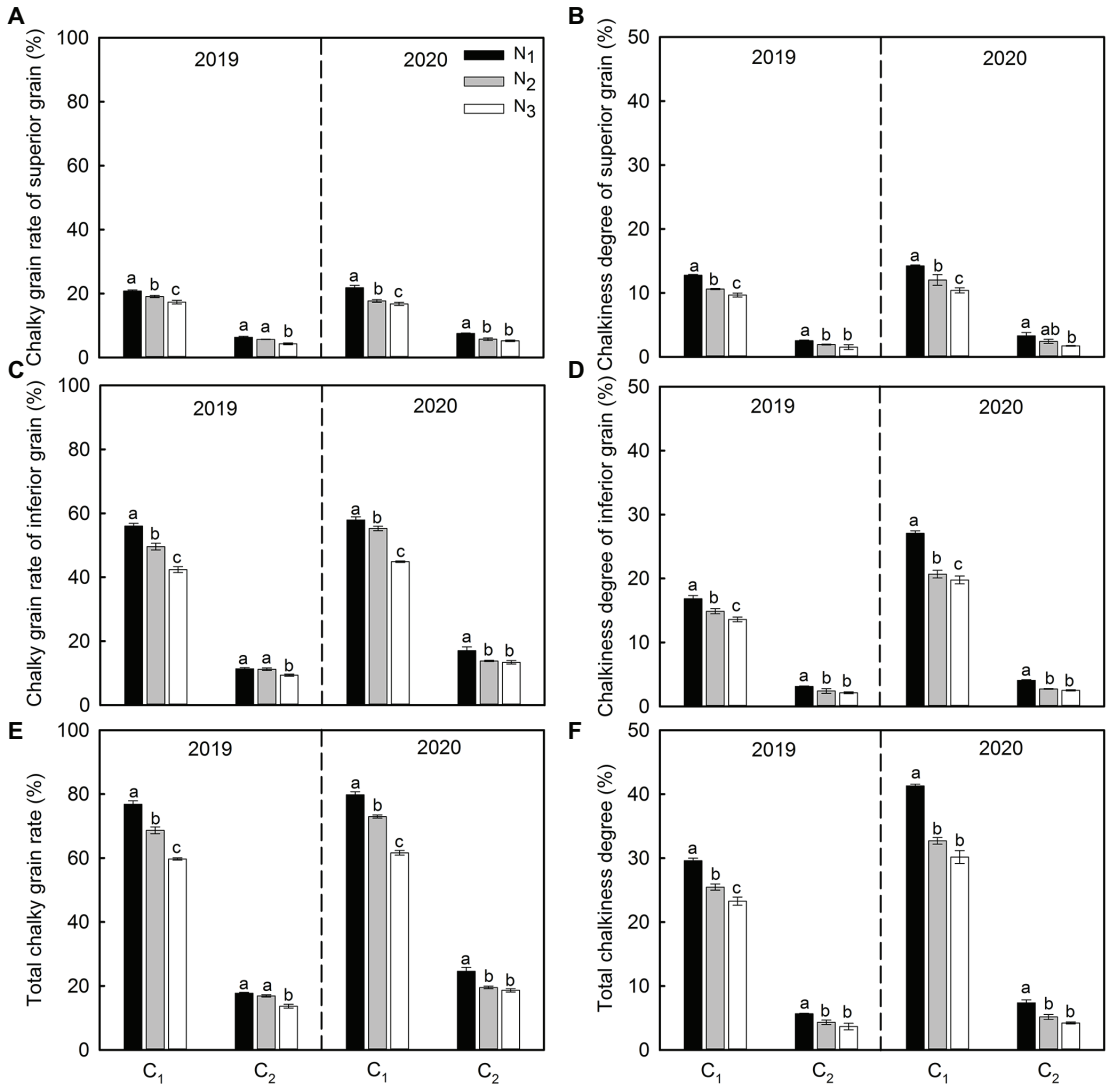
Changes of chalky grain rate **(A,C)** and chalkiness degree **(B,D)** of superior and inferior grains and changes of total chalky grain rate **(E)** and chalkiness degree **(F)** under different N application rates. Vertical bars represent ±SE of the mean (*n* = 3). Different lowercase letters indicate the statistical difference between treatments at the 0.05 level according to the LSD test. C_1_: Chuannongyou 508; C_2_: Shuangyou 573; N_1_: 75 kg ha^−1^; N_2_: 150 kg ha^−1^; N_3_: 225 kg ha^−1^.

N application rates significantly affected the formation and distribution of chalkiness characteristics. Chalky grain rate and chalkiness degree of high and low chalkiness varieties were mostly concentrated in the inferior grains (Figure 2). In addition, the chalky grain rate and chalkiness degree of superior and inferior grains decreased significantly with increasing N application. After averaging the data for 2 years, compared with N_1_ treatment, the chalky grain rate and chalkiness degree of superior grains of C_1_ and C_2_ decreased by 13.8%–20.0%, 16.2%–25.7%, 17.8%–31.3%, and 25.1%–44.0%, respectively, at N_2_ and N_3_ levels, whereas the chalky grain rate and chalkiness degree of inferior grains decreased by 8.0%–23.4%, 19.1%–24.0%, 11.9%–19.8%, and 28.7%–35.7% (Figures 2A–D). This eventually led to the overall decline of chalky grain rate and chalkiness degree (Figures 2E–F).

### Dynamic Accumulation of Carbohydrates in Functional Leaves

**Figure 3:**
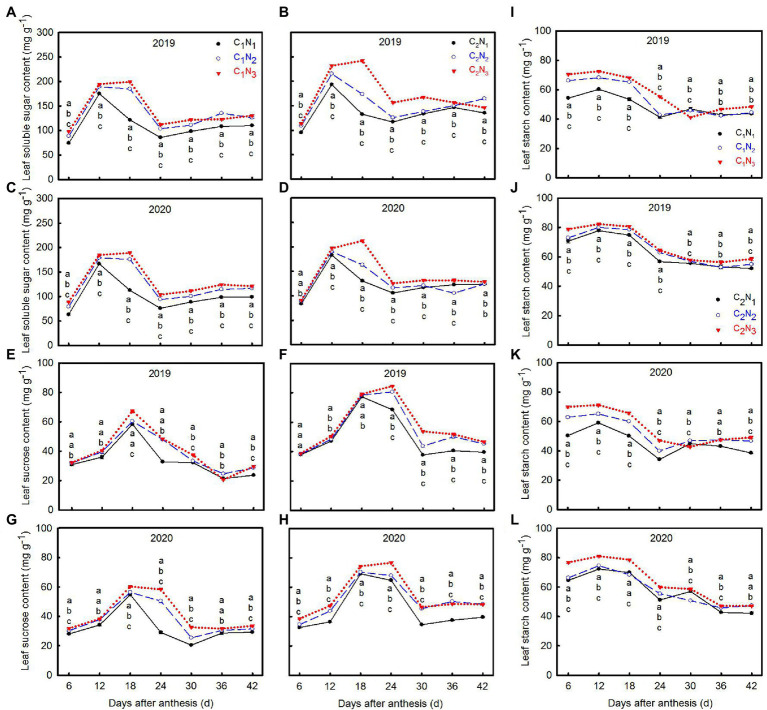
Changes of soluble sugar **(A–D)**, sucrose **(E–H)**, and starch **(I–L)** contents in top three leaves at 6, 12, 18, 24, 30, 36, and 42 days after anthesis under different N application rates. Data are mean ± SE (*n* = 3). Different lowercase letters from top to bottom indicate statistical difference between treatments at the 0.05 level according to the LSD test. C_1_: Chuannongyou 508; C_2_: Shuangyou 573; N_1_: 75 kg ha^−1^; N_2_: 150 kg ha^−1^; N_3_: 225 kg ha^−1^.

There was a significant difference in the carbohydrate content of the functional leaves under three N application levels (Figure 3). With increasing N application, the contents of soluble sugar, sucrose, and starch content in functional leaves increased significantly (Figures 3A–L), and the carbohydrate synthesis ability of the C_2_ functional leaves was significantly better than that of C_1_. In addition, the soluble sugar in the functional leaves of high and low chalkiness varieties reached the peak at 12 DAA under N_1_ and N_2_ treatment, and extended to 18 DAA under N_3_ treatment (Figures 3A–D). Sucrose of C_1_ accumulated most at 18 DAA, while C_2_ accumulated at 24 DAA (except C_2_N_1_) (Figures 3E–H). Except for starch content, the other sugar contents rose initially and then declined during growth and finally tended to be flat. Under various N application rates, the time periods of rapid accumulation of soluble sugar and sucrose was 6–24 DAA (Figures 3A–H). There were some differences between the cultivars. Moreover, the starch content of the two cultivars decreased gradually as the functional leaves grew (Figures 3I–L).

### Dynamic Accumulation of Carbohydrate in Grains

**Figure 4:**
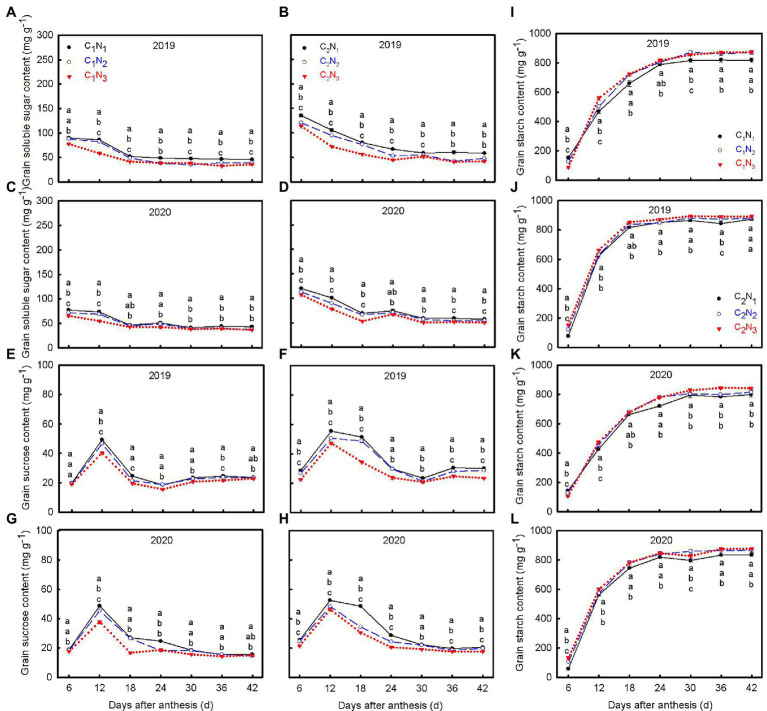
Changes of soluble sugar **(A–D)**, sucrose **(E–H)**, and starch **(I–L)** contents in grains at 6, 12, 18, 24, 30, 36, and 42 days after anthesis under different N application rates. Data are mean ± SE (*n* = 3). Different lowercase letters from top to bottom indicate statistical difference between treatments at the 0.05 level according to the LSD test. C_1_: Chuannongyou 508; C_2_: Shuangyou 573; N_1_: 75 kg ha^−1^; N_2_: 150 kg ha^−1^; N_3_: 225 kg ha^−1^.

The amount of N fertilizer significantly affected carbohydrate accumulation of grain (Figure 4). With the increase in N application, the soluble sugar and sucrose content in grains decreased significantly, while the starch content increased (Figures 4A–L). At the same time, the carbohydrate synthesis and accumulation capacity in C_2_ grains were more substantial than that in C_1_ grains. It is worth noting that soluble sugar gradually decreased with increasing post-anthesis time (6–30 DAA) and then stabilized (Figures 4A–D). The descending order was N_1_, N_2_, and N_3_. In addition, except for C_1_N_1_, sucrose reached a peak at 12 DAA and then decreased slowly (Figures 4E–H). In terms of starch content, with increasing post-anthesis time, starch accumulation gradually increased till it was stable (Figures 4I–L). At 6–12 DAA, starch accumulation in C_2_ was more rapid than that in C_1_.

### Grain Shape Parameters

**Figure 5:**
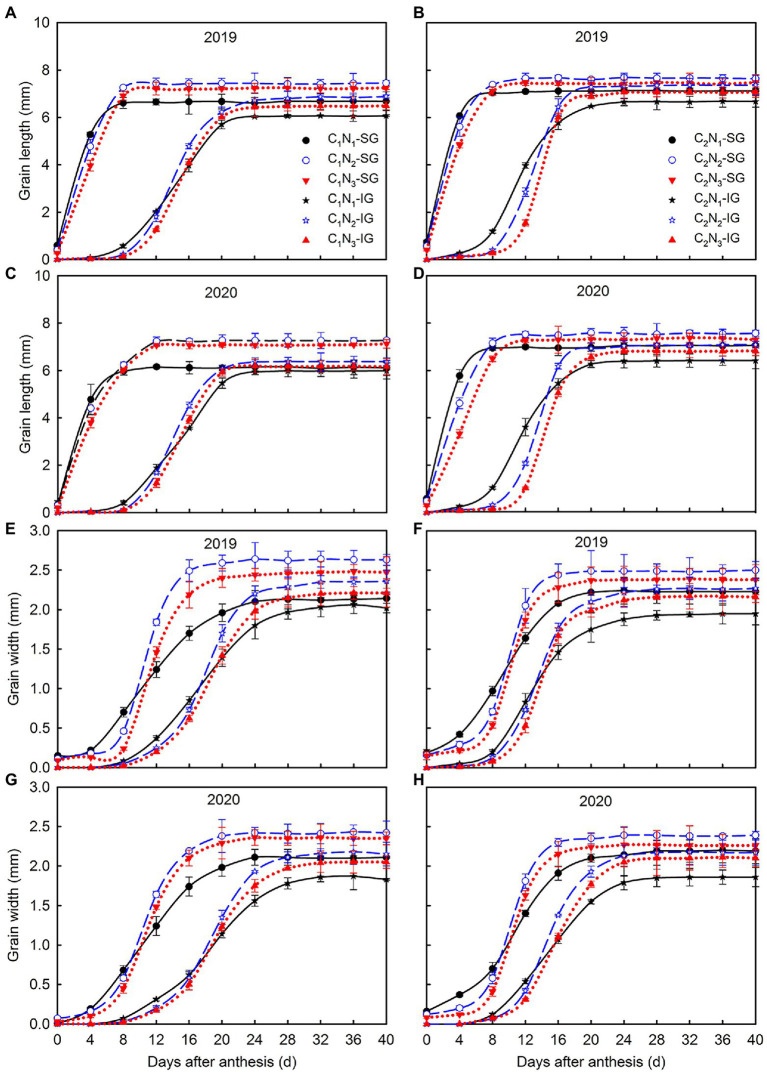
Changes of length **(A–D)** and width **(E–H)** of superior and inferior grains under different N application rates. Data are mean ± SE (*n* = 3). SG: superior grain; IG: inferior grain; C_1_: Chuannongyou 508; C_2_: Shuangyou 573; N_1_: 75 kg ha^−1^; N_2_: 150 kg ha^−1^; N_3_: 225 kg ha^−1^.

Size is a key factor affecting the grain’s ability to accept carbohydrates (Figure 5). In the mature stage, the length and width of high and low chalkiness varieties’ superior and inferior grains decreased in the following order, N_2_, N_3_, and N_1_ under each N treatment. The ovary of superior grains elongated lengthwise to its full length at 6–8 DAA at the N_1_ level, while at N_2_ and N_3_ levels, it extended to its full size at 8–12 DAA. The ovary development of inferior grains was completed 24–28 DAA. Under all N treatments, C_2_ superior and inferior grains developed faster than C_1_ (Figures 5A–D). After that, the development in width becomes evident. The full width of superior and inferior grains was reached at 20–24 and 28–32 DAA (Figures 5E–H), respectively.

### Grain Filling Characteristics in Early, Middle, and Late Growth Stage

**Figure 6:**
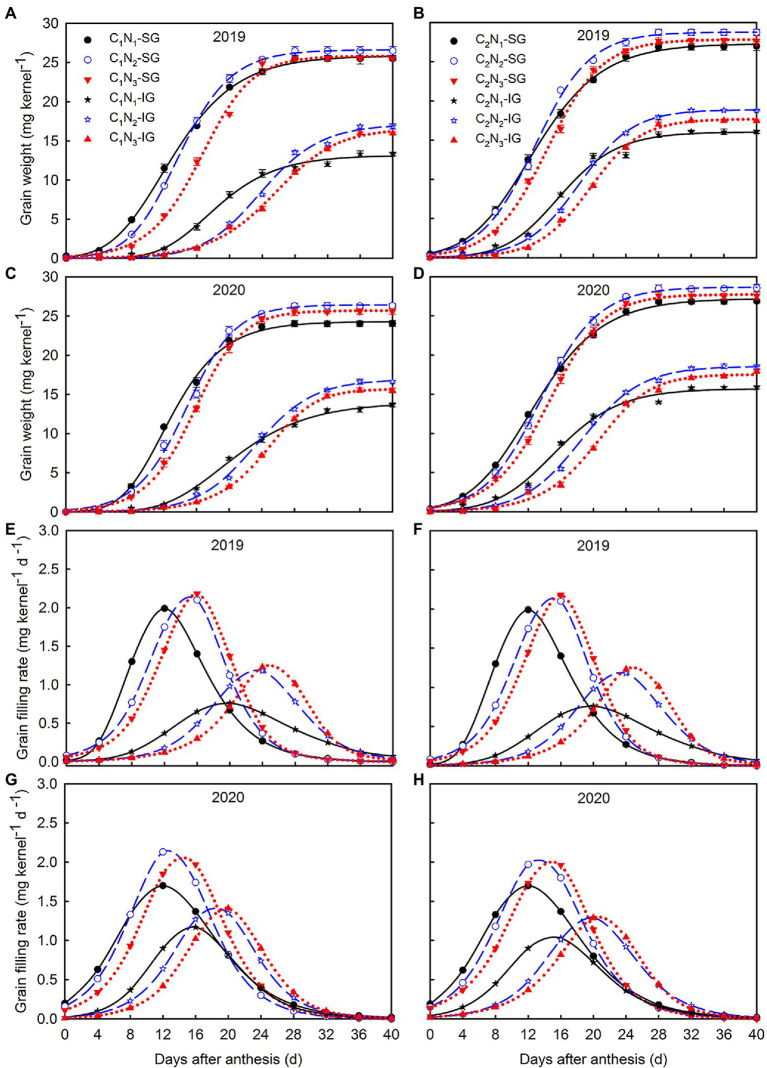
Changes of weight grain **(A–D)** and filling rate **(E–H)** of superior and inferior grains under different N application rates. Data are mean ± SE (*n* = 3). SG: superior grain; IG: inferior grain; C_1_: Chuannongyou 508; C_2_: Shuangyou 573; N_1_: 75 kg ha^−1^; N_2_: 150 kg ha^−1^; N_3_: 225 kg ha^−1^.

The dynamic changes in weight gain of the superior and inferior grains after anthesis of high and low chalkiness varieties under different N application rates conformed to the Richards equation, with the coefficients of determination above 0.99 (Table 2; Figure 6). The A value of the superior and inferior grains first increased and then decreased, and R_0_ decreased as N rates increased. Meanwhile, GR_max_ and GR_mean_ of the superior and inferior grains of the two varieties improved in N_2_ and N_3_ compared with N_1_ (Table 2; Figures 6E–H). Additionally, the T_max_ and W_max_ of the superior grains and inferior grains (except C_2_ in 2019) increased with increasing N fertilizer application. This result indicates that increasing N fertilizer application can promote grain filling rate and shorten the time required to attain the maximum filling rate (Table 2; Figures 6A–D). Meanwhile, despite the short grain filling period, MGR_m_ and RGC_m_ of the superior and inferior grains in this stage remained the highest (Table 3). With the increase in N application, days in the early stages of grain filling and RGC_e_ increased, while days in the middle and late stage of grain filling, RGC_m_ and RGC_L_ decreased. Moreover, compared to the N_1_ treatment, the average grain filling rate of superior and inferior grains at each stage was increased under N_2_ and N_3_ treatments. Compared with C_1_, C_2_ superior and inferior grains had shorter grain filling times, higher average grain filling rate and contribution rate.

**Table 2:** Effects of nitrogen application rate on parameters of Richard equation and grain filling parameters.

Years	Cultivars	Grain position	N application	Parameters of Richard equation					Grain filling parameters				
				*R* ^2^	*A* (mg·kernel^−1^)	*B*	*K*	*N*	R_0_	T_max_ (mg·kernel^−1^)	W_max_ (mg·kernel^−1^)	GR_max_ (mg·kernel^−1^)	GR_mean_ (mg·kernel^−1^)
2019	C_1_	Superior grain	N_1_	0.9992	25.86	3.053	0.2046	0.2788	0.734	11.70	10.70	1.71	1.16
			N_2_	0.9999	26.61	26.91	0.2833	0.6083	0.466	13.38	12.18	2.15	1.45
			N_3_	0.9976	25.82	330.0	0.3353	1.2078	0.278	16.73	13.40	2.04	1.35
		Inferior grain	N_1_	0.9967	13.17	11.46	0.2183	0.2649	0.824	17.26	5.42	0.94	0.63
			N_2_	0.9972	17.12	347.6	0.2585	0.7753	0.333	23.62	8.17	1.19	0.80
			N_3_	0.9981	16.55	1,661	0.2784	1.2467	0.223	25.84	8.65	1.07	0.71
	C_2_	Superior grain	N_1_	0.9994	27.30	6.679	0.2121	0.5286	0.401	11.96	12.23	1.70	1.14
			N_2_	0.9986	28.78	38.95	0.2929	0.9159	0.320	12.80	14.15	2.16	1.45
			N_3_	0.9988	27.84	72.07	0.2989	0.9829	0.304	14.37	13.87	2.09	1.39
		Inferior grain	N_1_	0.9927	16.05	37.04	0.2607	0.644	0.405	15.54	7.42	1.18	0.79
			N_2_	0.9996	18.89	279.1	0.3063	1.0168	0.301	18.33	9.48	1.44	0.96
			N_3_	0.9989	17.70	626.6	0.3246	1.0593	0.306	19.66	8.95	1.41	0.94
2020	C_1_	Superior grain	N_1_	0.9986	24.29	4.956	0.2506	0.2573	0.974	11.80	9.98	1.99	1.35
			N_2_	0.9982	26.43	176.9	0.3406	1.1147	0.306	14.88	13.50	2.17	1.45
			N_3_	0.9995	25.69	401.2	0.3631	1.2243	0.297	15.95	13.37	2.18	1.45
		Inferior grain	N_1_	0.9979	14.11	9.960	0.1714	0.3468	0.494	19.59	5.98	0.76	0.52
			N_2_	0.9986	16.90	615.0	0.2789	0.9637	0.289	23.16	8.39	1.19	0.80
			N_3_	0.9998	15.70	28,227	0.3875	1.6214	0.239	25.20	8.67	1.28	0.84
	C_2_	Superior grain	N_1_	0.9994	27.21	6.006	0.2103	0.4908	0.428	11.91	12.06	1.70	1.15
			N_2_	0.9997	28.68	35.14	0.2756	0.8769	0.314	13.39	13.99	2.05	1.37
			N_3_	0.9994	27.73	118.4	0.3108	1.2111	0.257	14.74	14.40	2.02	1.34
		Inferior grain	N_1_	0.9944	15.71	20.56	0.2351	0.6132	0.383	14.94	7.20	1.05	0.71
			N_2_	0.9984	18.58	187.9	0.2708	0.9216	0.294	19.64	9.15	1.29	0.86
			N_3_	0.9964	17.54	1,549	0.3360	1.4003	0.240	20.86	9.39	1.31	0.87

*Data are the fitting data after three repeated averages. *R*^2^: fitting degree; A: final weight of a kernel; B: initial parameter; k: growth rate parameter; N: shape parameter; R_0_: initial growth potential of grain; T_max_: days required for grains to reach the maximum filling rate; W_max_: weight of a kernel at maximum filling rate; GR_max_: maximum grain filling rate; GR_mean_: mean grain filling rate; C_1_: Chuannongyou 508; C_2_: Shuangyou 573; N_1_: 75 kg ha^−1^; N_2_: 150 kg ha^−1^; and N_3_: 225 kg ha^−1^*.

**Table 3:** Effects of nitrogen application rate on grain filling characteristics in early, middle, and late stages.

Years	Cultivars	Grain position	*N* application	Early stage			Middle stage			Late stage		
				Days (d)	MGR_e_ (mg·kernel^−1^·d^−1^)	RGC_e_ (%)	Days (d)	MGR_m_ (mg·kernel^−1^·d^−1^)	RGC_m_ (%)	Days (d)	MGR_l_ (mg·kernel^−1^·d^−1^)	RGC_l_ (%)
2019	C_1_	Superior grain	N_1_	6.43	0.47	11.69	10.54	1.49	60.55	17.21	0.45	26.76
			N_2_	9.16	0.47	16.33	8.44	1.87	59.42	12.01	0.56	23.25
			N_3_	12.63	0.48	23.41	8.20	1.79	56.79	9.60	0.45	18.80
		Inferior grain	N_1_	12.34	0.12	11.48	9.83	0.81	60.59	16.15	0.21	26.93
			N_2_	18.79	0.17	18.47	9.67	1.04	58.73	12.95	0.19	21.81
			N_3_	20.87	0.19	23.82	9.95	0.94	56.61	11.52	0.23	18.57
	C_2_	Superior grain	N_1_	6.45	0.65	15.26	11.02	1.48	59.73	16.17	0.48	24.00
			N_2_	8.39	0.69	20.16	8.82	1.90	58.11	11.28	0.49	20.73
			N_3_	9.98	0.58	20.94	8.78	1.83	57.81	10.98	0.45	20.25
		Inferior grain	N_1_	10.91	0.25	16.80	9.26	1.03	59.28	13.00	0.22	22.92
			N_2_	14.02	0.29	21.32	8.63	1.26	57.66	10.69	0.22	20.02
			N_3_	15.55	0.25	21.80	8.22	1.24	57.47	10.05	0.27	19.73
2020	C_1_	Superior grain	N_1_	7.54	0.37	11.37	8.54	1.72	60.61	14.08	0.47	27.02
			N_2_	10.91	0.54	22.41	7.92	1.91	57.22	9.53	0.54	19.37
			N_3_	12.15	0.50	23.58	7.60	1.92	56.72	8.85	0.54	18.70
		Inferior grain	N_1_	13.15	0.14	12.70	12.88	0.66	60.36	20.39	0.18	25.94
			N_2_	18.47	0.19	20.72	9.37	1.04	57.90	11.79	0.29	20.38
			N_3_	21.38	0.20	27.47	7.64	1.13	54.92	8.03	0.32	16.61
	C_2_	Superior grain	N_1_	6.41	0.63	14.75	10.99	1.48	59.87	16.37	0.41	24.38
			N_2_	8.74	0.65	19.70	9.29	1.80	58.28	12.03	0.50	21.02
			N_3_	10.32	0.63	23.44	8.85	1.78	56.78	10.35	0.50	18.78
		Inferior grain	N_1_	9.85	0.26	16.40	10.19	0.92	59.40	14.46	0.25	23.20
			N_2_	14.86	0.25	20.23	9.56	1.13	58.09	12.19	0.32	20.69
			N_3_	16.62	0.27	25.37	8.49	1.16	55.91	9.43	0.33	17.71

*Data are the fitting data after three repeated averages. MGR_e_: mean grain filling rate in early stage; MGR_m_: mean grain filling rate in medium stage; MGR_l_: mean grain filling rate in late stage; RGC_e_: contribution of early grain filling rate to final grain weight; RGC_m_: contribution of medium grain filling rate to final grain weight; RGC_l_: contribution of late grain filling rate to final grain weight; C_1_: Chuannongyou 508; C_2_: Shuangyou 573; N_1_: 75 kg ha^−1^; N_2_: 150 kg ha^−1^; and N_3_: 225 kg ha^−1^*.

### Principal Component Analysis of Grain Chalkiness Traits With Grain Filling Parameters or Carbohydrate Accumulation

**Figure 7:**
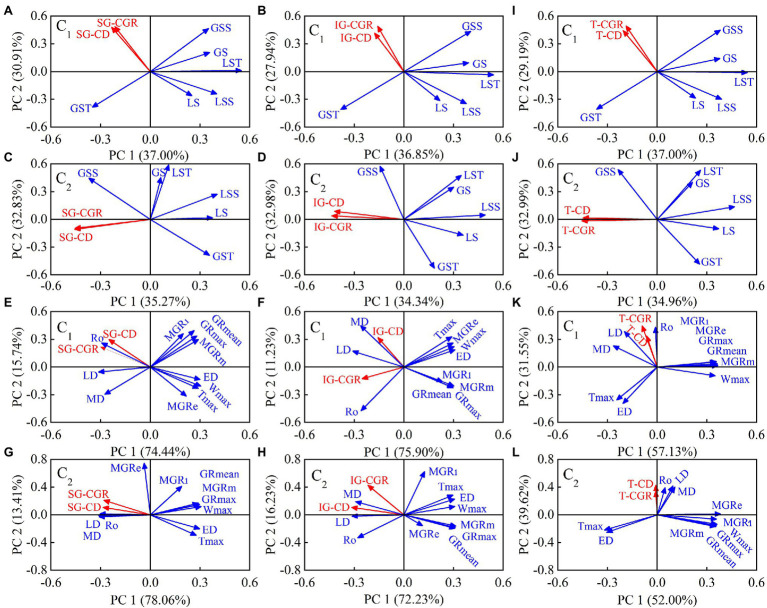
Principal component analysis of chalky grain rate and chalkiness degree of superior and inferior grains **(A–H)**, as well as the total chalky grain rate and chalkiness degree **(I–L)** with carbohydrate or grain filling parameters under different N application rates, and the vectors represent the loading scores of variables for PC1 and PC2. PC1: principal component 1; PC2: principal component 2; C_1_: Chuannongyou 508; C_2_: Shuangyou 573; SG-CGR: superior grain chalky grain rate; SG-CD: superior grain chalkiness degree; IG-CGR: inferior grain chalky grain rate; IG-CD: inferior grain chalkiness degree; T-CGR: total chalky grain rate; T-CD: total chalkiness degree; GSS: grain soluble sugar; GS: grain sucrose; GST: grain starch; LSS: leaf soluble sugar; LS: leaf sucrose; LST: leaf starch; R_0_: initial growth potential of grain; T_max_: days required for grains to reach the maximum filling rate; W_max_: weight of a kernel at maximum filling rate; GR_max_: maximum grain filling rate; GR_mean_: mean grain filling rate; MGR_e_: Mean grain filling rate in early stage; MGR_m_: Mean grain filling rate in medium stage; MGR_l_: Mean grain filling rate in late stage; ED: days of early stage grain filling; MD: days of middle stage grain filling; LD: days of late stage grain filling.

Under different N application rates, the principal components 1 and 2 of C_1_ explained 67.9%, 64.8%, and 66.2% of the total variation in superior grain chalky grain rate and chalkiness degree, inferior grain chalky grain rate and chalkiness degree, and total chalky grain rate and chalkiness degree, respectively (Figures 7A,B,I), while principal components 1 and 2 of C_2_ explained 68.1%, 67.3%, and 68.0% of the total variation, respectively (Figures 7C,D,J). The relationship between these variables suggested that chalkiness formation was negatively correlated with soluble sugar and sucrose contents in functional leaves and sucrose content in grains. Concerning the grain filling parameters, principal components 1 and 2 of C_1_ accounted for 90.2%, 87.1%, and 88.7% of the total variance, respectively (Figures 7E,F,K), while principal components 1 and 2 of C_2_ accounted for 91.5%, 88.5%, and 91.6% of the total variance, respectively (Figures 7G,H,L). The load scores of the principal component analysis indicated that the formation of superior and inferior grains chalky grain rate and chalkiness degree were closely related to grain filling. This eventually led to a positive correlation between total chalky grain rate and chalkiness with days in the middle and later stages of grain filling, R_0_, RGC_m_ and RGC_l_, while a negative association with A, W_max_, MGR_e_, MGR_m_, MGR_l_, GR_max_, and GR_mean_. In contrast, the dynamic accumulation of carbohydrates in functional leaves and grains of C_2_ and grain filling parameters explain chalkiness formation more fully than C_1_.

## Discussion

### Accumulation of Carbohydrates Affected the Formation of Chalkiness Quality

Rice chalkiness formation is a complex process controlled by the genotype and affected by cultivation environments (Phan et al., 2013; Zhao et al., 2020). It is common to address the mechanism of chalkiness formation from the perspective of genes. For example, Wang et al. (2008) showed that *G1F1* could encode cell wall sucrose transporter and regulate the distribution of carbon in the early stage of grain filling. Its absence will lead to the formation of chalkiness. Similarly, Satoh et al. (2008) pointed out that in the *PHO1* deletion mutant, the grain shrinks, the internal total starch content is significantly reduced, and the starch granules are small and oval, resulting in the white-core phenotype of the endosperm. However, the internal response mechanism and regulatory pathways due to cultivation practices, especially rice chalkiness to N fertilizer dosage and operation research mode, are still vague. In this study, the appropriate application of N fertilizer significantly decreased chalky grain rate and chalkiness degree of the superior grains and inferior grains and then interfered with increasing total chalky grain rate and chalkiness degree (Figures 2A–F), consistent with the conclusions of previous studies (Zhou et al., 2015). However, excessive N application would increase the chalkiness traits (Wei et al., 2018a). In addition, this study found that the chalkiness characteristics of rice mainly were concentrated in the inferior grains (Figures 2C,D).

In rice source-sink theory, source refers to functional leaves and leaf sheaths. Sink mainly means grains. The assimilates produced in the functional leaves through photosynthesis are the key to ensuring grain filling and rice quality formation (Ariovich and Cresswell, 1983). The potential mechanism of how these assimilates influence chalkiness formation from source to sink needs to be further explored. Previous studies have demonstrated that high N utilization can promote the transfer of assimilates from functional leaves to grains (Ariovich and Cresswell, 1983). In harsh environments, such as high temperature and drought stress, N supply can regulate the photosynthesis of functional leaves and the accumulation of starch in grains to reduce chalkiness formation (Zhang et al., 2008; Tang et al., 2019). This study showed that the contents of soluble sugar, sucrose, fructose, glucose, and starch in the functional leaves increased significantly as N rates increased (Figures 3G–L; Supplementary Figures 1A–H). Except for starch, they increased first and then decreased as days after anthesis. Among them, soluble sugar and sucrose were the most relevant to the chalkiness characteristics of rice (Figures 7A–D,I,J). This indicates that increasing N fertilizer promoted the synthesis of carbohydrates in functional leaves, especially the accumulation of soluble sugar and sucrose, which were transported to grains through stems and sheaths, accelerated grain filling and material distribution, and was conducive to the reduction of rice chalkiness.

Grain filling is a significant carbon metabolism process dominated by sucrose decomposition and starch synthesis. Currently, research on chalkiness formation mainly focuses on the activity of critical enzymes of carbon metabolism, gene expression, and the accumulation of assimilates (Fitzgerald et al., 2009; Ito et al., 2009; Lin et al., 2016, 2017; Tao et al., 2022). Soluble sugar, starch, protein, and other forms of assimilates (e.g., sucrose) and their relationship in grains significantly impact rice chalkiness (Chen et al., 2021). An appropriate amount of N fertilizer is conducive to the normal development of starch granules (Zhou et al., 2020). Storage proteins fill the spaces between the starch granules, decrease the gap between them, and make the arrangement of starch granules denser, thus reducing chalkiness (Wada et al., 2019). Some studies also believed that a higher level of soluble sugar in grains could enhance the compactness of protein subunits of protein bodies, supporting the accumulation of storage protein and then reducing the tightness degree of rice (Yu et al., 2016). In this study, with increasing N application, the ability of grain to synthesize starch increased, and promoted the transformation of soluble sugar to starch, resulting in a lower content of soluble sugar and a higher level of starch in the later stage of grain filling (30–42 DAA). This may reduce the polymerization ability between starch, protein, and lipid to improve the composite strength of starch granules and reduce the chalkiness of rice. These results differ marginally from the results reported by Wada et al. (2019) and Yu et al. (2016).

### Relationship Between Grain Filling Characteristics and Rice Chalkiness Formation

N regulation significantly affects the development and grain filling of superior and inferior grains to determine the formation of rice chalkiness. Rice grain filling is a process of dynamic synthesis and accumulation. The length, width, water content, and filling parameters of grains are closely related to chalkiness formation (Gong et al., 2017; Sun et al., 2022). The length and width of the grain reflect the ability of rice grains to accept carbohydrates. In our study, the length and width of the superior and inferior grain accelerated and increased under N_2_ and N_3_ conditions compared with N_1_(Figures 5A–H), which was conducive to improving the ability of grains to accept carbohydrates and effectively alleviate the contradiction between source and sink, thereby reducing the occurrence of chalkiness (Figures 2A–F). Further, under the condition of N_1_, the filling rate of grains decreases, resulting in the rapid reduction of water content, premature water loss and precocity of grain, affecting the synthesis and accumulation of starch and then making its chalkiness relatively higher.

Indeed, grain filling parameters (e.g., R_0_, T_max_, W_max_, GR_max_, and GR_mean_) and grain filling differences at each stage are crucial for a macro study of chalkiness development (Bridgemohan and Bridgemohan, 2014; Dou et al., 2017). Previous comparative studies between varieties found that chalkiness and grain filling dynamic parameters generally showed an upward parabolic relationship (Zhang et al., 2021). It is possible to reduce the chalkiness by optimizing the grain filling process with appropriate cultivation control measures. In the present study, with increasing N application, R_0_ of superior and inferior grain decreased, while GR_max_ and GR_mean_ increased to shorten the T_max_ required to reach W_max_. The principal component analysis also showed that total chalky grain rate and chalkiness degree were negatively correlated with GR_max_ and GR_mean_, indicating that increasing N fertilizer optimized grain filling in the whole filling stage and then reduced total chalky grain rate and chalkiness degree. This may be related to optimizing leaf carbohydrate accumulation ability and essential carbon and nitrogen metabolism (e.g., ADP-Glucose pyrophosphorylase) (Xi et al., 2020). Theoretically, the grains obtained more carbohydrates in the early grain filling stage. The physiological and metabolic pathways became very active, which may significantly impact chalkiness formation (Cai et al., 2006). However, this study showed that the whole grain filling stage would affect chalkiness formation. In particular, increased N accelerated the grain filling of inferior grain in the middle and later stages, conducive to chalkiness reduction.

Moreover, the chalkiness decreased when the superior and inferior grains were filled simultaneously and increased when the superior and inferior grains were grouted asynchronous (Shimoyanagi et al., 2021; Sun et al., 2022). In this study, Chalky grain rate and chalkiness degree in the two cultivars were mainly distributed in the inferior grains and decreased as N rates increased, further decreasing total chalky grain rate and chalkiness degree. Increasing N fertilizer promotes the synthesis and distribution of carbohydrates in the functional leaves, and the superior grain obtained carbohydrates preferentially. After rapid grain filling, the carbohydrates were quickly distributed to the inferior grain to enhance their grouting and maturity, reducing their chalkiness. On the contrary, the superior grain was supplied with carbohydrates slowly, and the inferior grain filling was inhibited, increasing chalkiness (Bridgemohan and Bridgemohan, 2014; Deng et al., 2018; Sun et al., 2022). Hence, grain position, grain size, grain filling speed, insufficient synthesis, or uneven distribution of carbohydrates are related to the formation of rice chalkiness.

## Conclusion

The present study revealed little difference in grain filling intensity of the superior grain in both C_1_ and C_2_. The vigorous and weak grain filling of the inferior grain may be an important reason for determining high and low chalkiness in rice. Furthermore, the dynamic accumulation of carbohydrate and grain filling characteristics in the functional leaves and grains under various N fertilizer treatments was closely associated with rice chalkiness formation. Moderate N application (i) improved the accumulation of carbohydrates in the functional leaves, especially the transport of soluble sugar to the grains, (ii) maintained the transformation of soluble sugar to starch in grains, (iii) accelerated the development of length and width of the superior and inferior grains, (iv) enhanced and optimized the grain filling of the superior and inferior grains, thereby contributing to the decreased total chalky grain rate and chalkiness degree. A practical N application could be a simple and easy way to achieve rice low chalkiness.

## Data Availability Statement

The original contributions presented in the study are included in the article/Supplementary Material, further inquiries can be directed to the corresponding authors.

## Author Contributions

CG contributed to mapping and writing—original draft. XY, FY, and KX performed the experiments and collected the data. YW and QZ contributed to the methodology. ZW, LH, and PF contributed to the formal analysis. ZY, ZC, and YS revised the manuscript. JM administered the project and provided funding acquisition. All authors contributed to the article and approved the submitted version.

## Funding

This work was supported by the Research Program Foundation of Key Laboratory of Sichuan Province, China, the Sichuan Provincial Science and Technology support projects (2020YJ0411 and 2021YJ0281), and the Rice Breeding Project Foundation of Sichuan Provincial Science and Technology Department (2021YFYZ0005).

## Conflict of Interest

The authors declare that the research was conducted in the absence of any commercial or financial relationships that could be construed as a potential conflict of interest.

## Publisher’s Note

All claims expressed in this article are solely those of the authors and do not necessarily represent those of their affiliated organizations, or those of the publisher, the editors and the reviewers. Any product that may be evaluated in this article, or claim that may be made by its manufacturer, is not guaranteed or endorsed by the publisher.
