# Supplementary material for: Nitrogen Application Rate Affects the Accumulation of Carbohydrates in Functional Leaves and Grains to Improve Grain Filling and Reduce the Occurrence of Chalkiness
Source: Front Plant Sci. 2022 Jun 24;13:921130. doi: 10.3389/fpls.2022.921130 (PMC9270005; doi:10.3389/fpls.2022.921130)
Supplement: Supplementary file 1 [file Data_Sheet_1.doc]

**Determination Items and Methods**

**Determination Method of Fructose and Glucose and Calculation Method of Grain Water Content**

The fructose content was determined by taking 0.5 mL of the testing solution, mixing it with one milliliter of H_2_O and 2 mL of 0.1 % resorcinol and reacting in a water bath at 80°C for 10 min. The absorbance was measured at 480 nm.

The glucose content was determined by taking a 4.0 mL enzyme mixture (0.1 mLof glucose oxidase, 10 mg of O-dianisidine and 10 mg of horseradish peroxidase dissolved in 100 mL of 0.1 M acetic acid buffer) and mixing it with 2 mL of the testing solution. After preincubation at 30°C for 5 min, 8.0 mL 10 M sulfuric acid was added to terminate the reaction. The absorbance was measured at 505 nm.

Grain water content (1)

**RESULTS**

**Fructose and Glucose in Leaves**

With the increase of N application, the contents of fructose and glucose in functional leaves increased, and rose initially and then declined during growth and finally tended to be flat. In addition, under different N application rates, fructose and glucose accumulated more at 12‒18 and 24‒30 DAA, respectively (**Figures 1A-H**).

**Fructose and Glucose in Grains**

With the increase of N application, the fructose content in grains decreased significantly, while the glucose content increased. Fructose decreased gradually with increasing post-anthesis time (6‒30 DAA) and then stabilized. Among them, the descending order was N_1_, N_2_, and N_3_. Glucose reached a peak at 12 DAA and then decreased slowly (**Figures 2A-H**).

**Grain Water Content**

With increasing time after anthesis, the water content of superior brown rice decreased slowly, while inferior brown rice increased first and then decreased. Compared with the N_1_ level, the water content of superior and inferior brown rice had obvious advantages 20 days before flowering and then dropped rapidly, even lower than that of other N treatments (**Figures 3A-D**).





FIGURE 1 Changes of fructose (A, B, C, and D) and glucose (E, F, G, and H) contents in top three leaves at 6, 12, 18, 24, 30, 36 and 42 days after anthesis under different N application rates. Data are mean ± S.E. (n=3). Different lowercase letters from top to bottom indicate statistical difference between treatments at the 0.05 level according to the LSD test. C_1_: Chuannongyou 508; C_2_: Shuangyou 573; N_1_: 75 kg ha^-1^; N_2_: 150 kg ha^-1^; N_3_: 225 kg ha^-1^.





FIGURE 2 Changes of fructose (A, B, C, and D) and glucose (E, F, G, and H) contents in grains at 6, 12, 18, 24, 30, 36 and 42 days after anthesis under different N application rates. Data are mean ± S.E. (n=3). Different lowercase letters from top to bottom indicate statistical difference between treatments at the 0.05 level according to the LSD test. C_1_: Chuannongyou 508; C_2_: Shuangyou 573; N_1_: 75 kg ha^-1^; N_2_: 150 kg ha^-1^; N_3_: 225 kg ha^-1^.


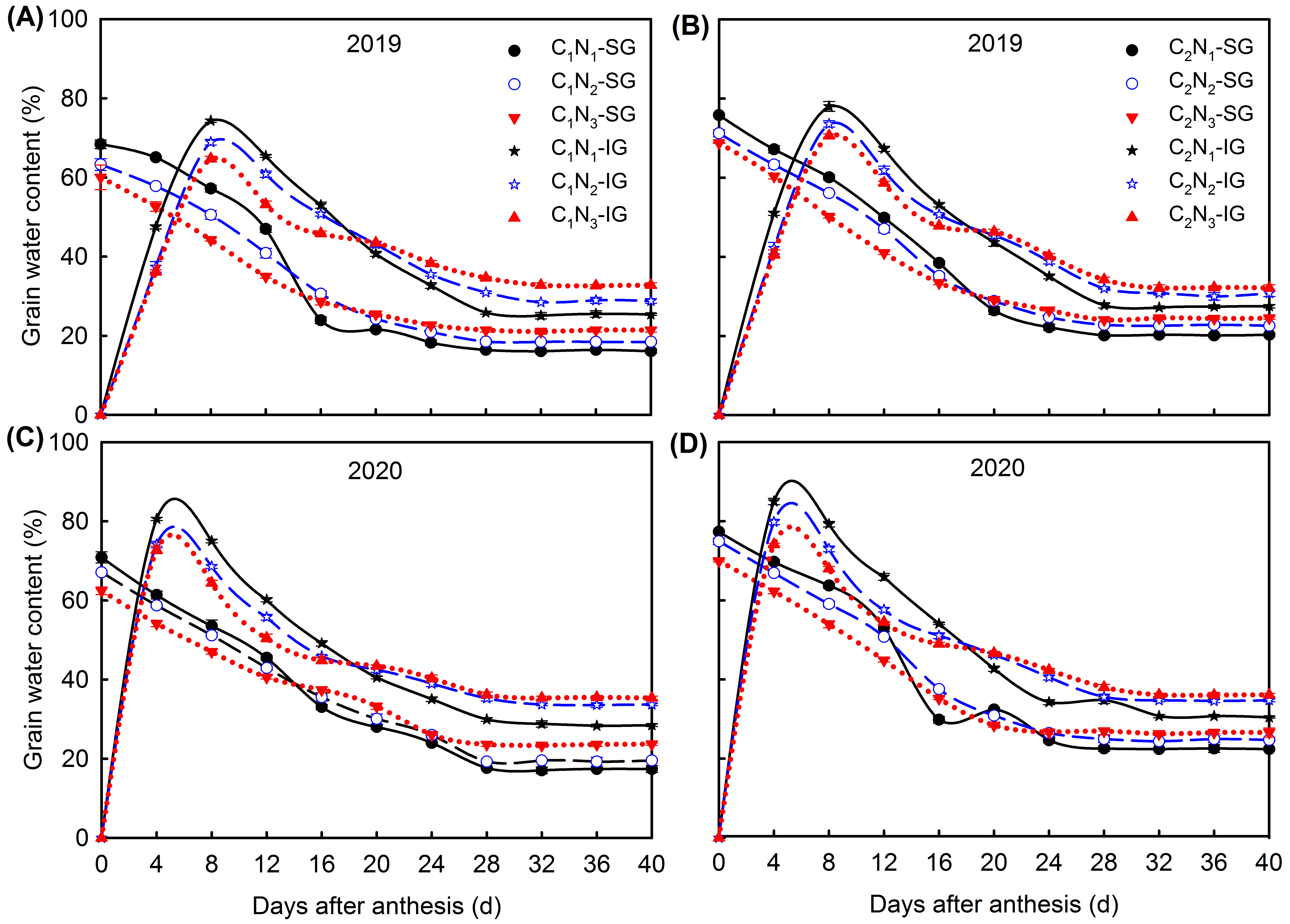


FIGURE 3 Changes of grain water (A, B, C, and D) content of superior and inferior grains under different N application rates. Data are mean ± S.E. (n=3). SG: superior grain; IG: inferior grain; C_1_: Chuannongyou 508; C_2_: Shuangyou 573; N_1_: 75 kg ha^-1^; N_2_: 150 kg ha^-1^; N_3_: 225 kg ha^-1^.
